# The Intestinal Microbiome Predicts Weight Loss on a Calorie-Restricted Diet and Is Associated With Improved Hepatic Steatosis

**DOI:** 10.3389/fnut.2021.718661

**Published:** 2021-07-08

**Authors:** Tien S. Dong, Kayti Luu, Venu Lagishetty, Farzaneh Sedighian, Shih-Lung Woo, Benjamin W. Dreskin, William Katzka, Candace Chang, Yi Zhou, Nerea Arias-Jayo, Julianne Yang, Aaron I. Ahdoot, Jason Ye, Zhaoping Li, Joseph R. Pisegna, Jonathan P. Jacobs

**Affiliations:** ^1^The Vatche and Tamar Manoukian Division of Digestive Diseases, Department of Medicine, David Geffen School of Medicine, University of California, Los Angeles, Los Angeles, CA, United States; ^2^UCLA Microbiome Center, David Geffen School of Medicine, University of California, Los Angeles, Los Angeles, CA, United States; ^3^Division of Gastroenterology, Hepatology, and Parenteral Nutrition, Veterans Administration Greater Los Angeles Healthcare System, Los Angeles, CA, United States; ^4^Department of Medicine, Veterans Administration Greater Los Angeles Healthcare System, Los Angeles, CA, United States; ^5^Center for Human Nutrition, David Geffen School of Medicine, University of California, Los Angeles, Los Angeles, CA, United States

**Keywords:** metabolic syndrome, metabolic associated fatty liver disease, microbiome, obesity, ultrasound elastography, controlled attenuated parameter

## Abstract

**Background:** The microbiome has been shown in pre-clinical and epidemiological studies to be important in both the development and treatment of obesity and metabolic associated fatty liver disease (MAFLD). However, few studies have examined the role of the microbiome in the clinical response to calorie restriction. To explore this area, we performed a prospective study examining the association of the intestinal microbiome with weight loss and change in hepatic steatosis on a calorie-restricted diet.

**Methods:** A prospective dietary intervention study of 80 overweight and obese participants was performed at the Greater West Los Angeles Veterans Affair Hospital. Patients were placed on a macronutrient standardized diet for 16 weeks, including 14 weeks of calorie restriction (500 calorie deficit). Body composition analysis by impedance, plasma lipid measurements, and ultrasound elastography to measure hepatic steatosis were performed at baseline and week 16. Intestinal microbiome composition was assessed using 16S rRNA gene sequencing. A per protocol analysis was performed on all subjects completing the trial (*n* = 46).

**Results:** Study completers showed significant reduction in weight, body mass index, total cholesterol, low density lipoprotein, and triglyceride. Subjects who lost at least 5% of their body weight had significantly greater reduction in serum triglyceride and hepatic steatosis than those with <5% body weight loss. *Enterococcus* and *Klebsiella* were reduced at the end of the trial while *Coprococcus* and *Collinsella* were increased. There were also significant baseline microbiome differences between patients who had at least 5% weight loss as compared to those that did not. *Lachnoclostridium* was positively associated with hepatic steatosis and *Actinomyces* was positively associated with hepatic steatosis and weight. Baseline microbiome profiles were able to predict which patients lost at least 5% of their body weight with an AUROC of 0.80.

**Conclusion:** Calorie restriction alters the intestinal microbiome and improves hepatic steatosis in those who experience significant weight loss. Baseline microbiome differences predict weight loss on a calorie–restricted diet and are associated with improvement in hepatic steatosis, suggesting a role of the gut microbiome in mediating the clinical response to calorie restriction.

## Introduction

Obesity has become a leading cause of morbidity and mortality in the United States, affecting an estimated 1 out of every 3–4 Americans (1, 2). The rise in the incidence of obesity has corresponded to a marked increase in obesity-related comorbidities such as cardiovascular disease, diabetes, and metabolic associated fatty liver disease (MAFLD) (3, 4). MAFLD, also commonly known as non-alcoholic fatty liver disease, is a disease that encompasses a spectrum that includes bland hepatic steatosis, steatohepatitis, and cirrhosis. With the increasing prevalence of obesity, it is estimated that MALFD will become the leading cause of cirrhosis and liver transplant in the next decade (5). Currently, there are no approved medications for the treatment of MAFLD and weight loss management is still the primary intervention for the treatment of MAFLD. Unfortunately, only 10–15% of patients are able to achieve significant enough weight loss with dietary and lifestyle modifications to affect MAFLD progression (6). Therefore it is important to understand the factors that affect weight loss in response to dietary interventions such as calorie-restricted diets.

Over the last decade, the intestinal microbiome has been discovered to be critical in the development and progression of obesity and MAFLD (7–9). For example, germ-free animals are resistant to weight gain as compared to normally housed mice (10). In pre-clinical models, the obese phenotype was transferrable to germ-free mice *via* the microbiome (11). Furthermore, it has been well-established that long-term dietary patterns are associated with differences in microbiome composition and function (12–14). Pre-clinical studies along with studies involving bariatric surgery in humans, have suggested that the gut microbiome may also play a role in response to weight loss interventions (15–17). However, there is relatively little human data on the association of the microbiome with response to calorie restriction diets. Studies have been published on short-term calorie restriction diets of 3–4 weeks duration, but not of longer duration diets required to achieve clinically significant weight loss (18, 19). To study the role of the microbiome in weight loss and improved hepatic steatosis in response to calorie restriction, we performed a prospective study of the microbiome of obese and overweight subjects placed on a calorie-restricted diet for 14 weeks.

## Materials and Methods

### Patient Recruitment

Subjects were recruited at the Greater West Los Angeles Veterans Affair Medical Center (ClinicalTrials.gov Identifier: NCT01146704). Inclusion criteria included age between 20 and 75 years old, BMI of at least 27 kg/m^2^, stable smoking habits for at least 6 months prior to enrollment or non-smoker and was willing to maintain those habits for the duration of the study. Exclusion criteria included significant weight change (>3.0 kg) in the month prior to enrollment, weight loss of >10 kg in the 6 months prior to enrollment, were already on a calorie-restricted diet within 4 months to enrollment, using any investigational drugs within 2 months, abnormal baseline labs (serum creatinine > 1.6 mg/dl; ALT, AST, total bilirubin > 2.0 times the upper limit of normal; triglycerides > 500 mg/dl, total cholesterol > 350 mg/dl, TSH outside of normal range), pregnant or planning to be pregnant, drinking more than 1 alcoholic beverage a day, prior bariatric surgery, personal history of malignancy or inflammatory bowel disease. All patients gave written and verbal consent. The study was approved by the Greater West Los Angeles Veterans Affair Medical Center Institutional Review Board.

### Study Design and Dietary Intervention

Age, sex, race, ethnicity, medical history, current medications, surgical history, smoking history, drinking history, were obtained at the time of enrollment. After enrollment, patients were randomized using a random number generator to one of two macronutrient standardized diets (30% fat, 15% protein, 55% carbohydrate, or 30% fat, 30% protein, 40% carbohydrate) then received dietary counseling with either a certified nutritionist or medical doctor. Patients were blinded to their diet allocation. The calorie restricted diet was implemented in two phases: (1) an initial macronutrient-standardized diet without calorie restriction for 2 weeks then the same macronutrient-standardized diet with a deficit of 500 calories from their calculated metabolic rate for 14 weeks. The subject's basal metabolic rate was calculated using the InBody Scanner (Cerritos, California, USA) body composition analysis adjusting for routine daily activity by multiplying the basal metabolic rate by 1.2. Subjects received dietary counseling during their baseline visit on their macronutrient standardized diet. After their initial 2 weeks, subjects were then further counseled on how to restrict their intake. Patients were instructed to log their meals on a daily basis using a standardized log sheet. Patients were followed every 2–4 weeks for further counseling on dietary intake until the end of the trial which occurred at 16 weeks post-enrollment. Ultrasound elastography was done at the initial baseline visit as well as the final visit using the FibroScan platform (Echosens, Waltham, MA, USA) with the XL probe by trained technician with at least 100 prior procedures (20). Blood was drawn at baseline and at the final visit and tested for total cholesterol, high density lipoprotein (HDL), low density lipoprotein (LDL), triglyceride, and hemoglobin A1c (HbA1c).

### Nutritional Assessment and Compliance

The Diet History Questionnaire III (DHQIII) was used to assess participants' baseline and final caloric intake and macronutrient composition. The DHQIII is a self-administered and validated diet questionnaire that asks patients about their food consumption over the previous month. It is supported by the National Cancer Institute and has been validated against other food frequency questionnaires (21). The DHQIII was administered at baseline and at the final visit. Compliance to a calorie restricted diet was defined as a reduction of 500 calories from their baseline food intake as measured by the DHQIII.

### Fecal Sampling and Processing

Subjects were given an at home stool kit for stool collection. Stool was collected in Para-Pak collection vials pre-filled with 95% ethanol, which allows stable storage for 2–4 weeks at room temperature (22). Patients were instructed to provide a stool sample within 24–48 h of their study visit. Patients were asked to return a sample at every visit, including the baseline and final visit. In total, 8 stool samples were collected from each participant. Received samples were then immediately stored in −80°C until processing. Samples were processed as a single batch. DNA from the stool was extracted using the ZymoBIOMICS DNA Microprep Kit (Zymo Research, Irvine, CA, USA) per the manufacturer's protocol. We sequenced the V4 hypervariable region of the 16S ribosomal DNA using the Illumina Novaseq 6000 as previously described (23). The raw sequences were processed using the DADA2 pipeline in R and taxonomy was assigned using the SILVA 132 database (24). The amplicon sequence variant (ASV) count table was then incorporated into QIIME2 (version 2020.11) for further analysis (25). Low abundant ASVs, those that were not present in at least 15% of samples, were removed from the analysis. The median sequence depth was 210,183 with a range from 92,624 to 427,748.

### Statistical Analysis

All analysis was done without knowing the patients' diet allocation. Clinical characteristics and outcomes were compared between baseline and the final visit at week 16 using the Wilcoxon signed-rank test. Clinical characteristics and outcomes between groups at baseline and at week 16 were compared using the Wilcoxon rank sum test. Hispanic ethnicity was incorporated as a race category for analysis. Significant weight loss was defined as having at least 5% weight change from baseline (26). Wilcoxon rank-sum test was performed to compare changes between those that had significant weight loss as compared to those that did not. Categorical data was compared using the Fisher's exact-test. Values are expressed as medians with their interquartile range (IQR). Microbiome data was analyzed using the same approaches as our previously published studies (27, 28). Beta diversity was determined using the robust Aitchison distance metric employed in QIIME2. This newer distance metric is able to discriminate better for human studies as compared to UniFrac or Bray-Curtis (29). Significance of beta diversity differences was determined using permutational multivariate analysis of variance as implemented in the “adonis” package in *R* (version 4.0.3, Vienna, Austria) controlling for subject and macronutrient diet group. Alpha diversity was calculated using the Shannon index, which measures species richness and evenness, with data rarefied to 92,623 sequences. Significance of differences in alpha diversity was calculated using repeated measures analysis of variance controlling for subject and macronutrient diet group. Differential abundance of microbial genera was assessed using DESeq2 in R which utilizes an empirical Bayesian approach to shrink dispersion and fit non-rarified count data to a negative binomial model (30). Differential abundance testing controlled for subject and macronutrient diet group. *P*-values were converted to q-values to correct for multiple hypothesis testing using a threshold of *q* < 0.05 for significance. The majority of patients who did not complete the study, dropped out of the study within the first 4 weeks. There were no clinical differences between the participants that did drop out vs. those that remained. Therefore, analysis was performed by a per protocol design, therefore excluding patients who did not complete the study. Using differentially abundant genera, a random forest classifier to predict significant weight loss and reduction in hepatic steatosis was created in *R* using the “randomForest” package with 1,001 trees and an optimized mtry similar to our previous published works (28). Features imputed into the model were those genera found to be differentially abundant. The accuracy of the model was estimated using 5-fold cross validation and presented as a receiver operating characteristic curve. Mediation analysis was performed using the “mediation” package in *R* with bootstrapping.

## Results

**Figure 1:**
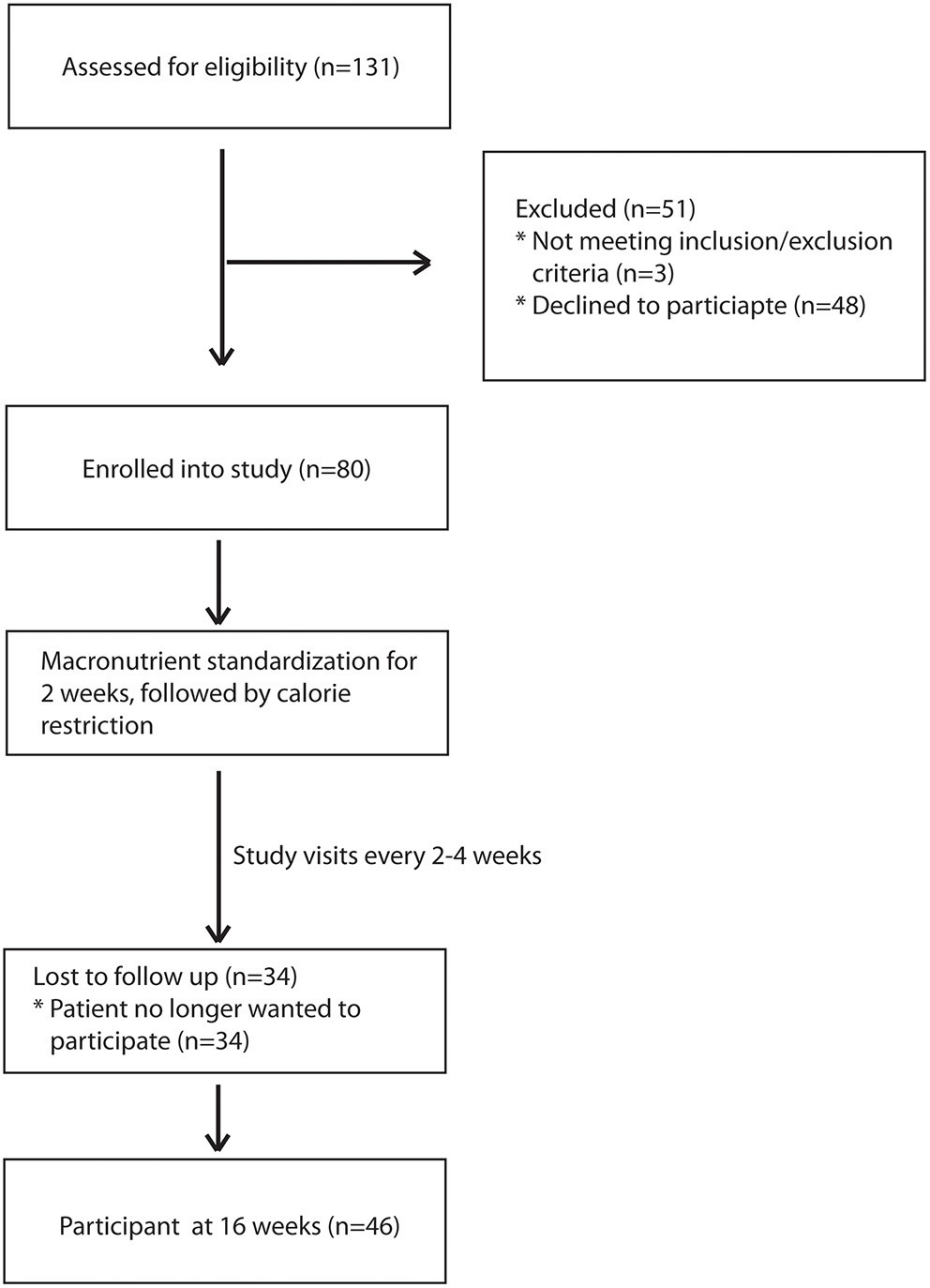
Study flow diagram.

During the recruitment period, 131 subjects were screened, and 80 subjects enrolled into the study (Figure 1). These subjects were placed on one of two macronutrient standardized diets varying in protein and carbohydrate intake for 2 weeks, then were transitioned to calorie-restricted diets with the same macronutrient profile (500 calories estimated deficit) for 14 weeks. Of the 80 enrolled subjects, 46 completed the trial. Given the moderate number of study completers and lack of a statistically significant difference in weight loss between the two macronutrient profiles (Supplementary Table 1), subjects in both macronutrient groups were combined to analyze the effects of calorie restriction. Patients who completed the trial had significant weight loss [102.3 kg (IQR 27.6) at baseline and 98.4 kg (IQR 27.3) at 16 weeks; *p* < 0.01] (Table 1). There was also a significant reduction in their BMI [34.3 kg/m^2^ (IQR 4.7) at baseline and 33.3 kg/m^2^ (IQR 6.3) at 16 weeks; *p* < 0.01]. In addition to weight loss, completers also had improvements in their total cholesterol [170.5 mg/dl (IQR 65.0) vs. 153 mg/dl (IQR 53.0); *p* < 0.01], HbA1c [6.0% (IQR 1.2) vs. 5.9 % (IQR 0.7); *p* = 0.04], and triglyceride [130 mg/dl (IQR 86) vs. 97 mg/dl (IQR 35); *p* < 0.01]. There was a trend in LDL improvement [98.5 mg/dl (IQR 54.0) vs. 80.0 mg/dl (IQR 53.3); *p* = 0.09] but there was no difference in HDL, hepatic fibrosis, or steatosis as measured by controlled attenuation parameter (CAP) score at 16 weeks as compared to baseline. At baseline, participants consumed a median of 2051.3 kcal while at the final visit, participants consumed a median of 1492.9 kcal (*p* < 0.01; Table 2). At the final visit, participants consumed significantly less fat (*p* < 0.01) and carbohydrate (*p* < 0.01) as compared to their baseline values as measured by the DHQIII. There was no difference in protein intake between the final visit and baseline visit (*p* = 0.11). While there was no change in steatosis from the final visit to the baseline visit, completers that achieved loss of at least 5% of body weight demonstrated a significant reduction in CAP score as compared to those that did not lose at least 5% of body weight [−65.5 dB/m (IQR 92.0) vs. 23.5 dB/m (IQR 72.0); *p* < 0.01; Table 3]. Similarly, patients who had at least 5% weight loss also had significantly greater improvement in serum triglycerides (*p* < 0.01) and fibrosis (*p* = 0.04). There were no differences by age, gender, or race between those that had significant weight loss vs. those that did not have significant weight loss. Furthermore, patients who lost significant weight did not have significant changes to their total calorie intake or macronutrient intake as compared to those that did not lose significant weight (Table 4). Twenty-five patients were deemed as compliant with the calorie restriction as determined by their DHQIII. There was no difference in the proportion of compliant patients between those that lost significant weight as compared to those that did not. In a subgroup analysis comparing participants with diabetes to those without diabetes, those without diabetes lost significantly more percent body weight as compared to those with diabetes [−8.6% (IQR 4.7) vs. −4.6% (IQR 5.3), *p* = 0.01].

**Table 1:** Patient characteristics, laboratory values, and ultrasound elastography measurements at baseline and at the end of the study (week 16).

	**Baseline (*n* = 46)**	**Week 16 (*n* = 46)**	***p*-value**
	**(*n* = 46)**	**(*n* = 46)**	
Gender (% males) (*n* = 35)	76.1	–	–
Age (years) (IQR)	57 (18)	–	–
BMI kg/m^2^ (IQR)	34.3 (4.7)	33.3 (6.3)	**<0.01**
Weight (kg) (IQR)	102.3 (27.6)	98.4 (27.3)	**<0.01**
HbA1c (IQR)	6.0 (1.2)	5.9 (0.7)	**0.04**
Total cholesterol (mg/dl) (IQR)	170.5 (65)	153 (53)	**<0.01**
HDL (mg/dl) (IQR)	42.9 (13.1)	42.5 (12.8)	0.83
LDL (mg/dl) (IQR)	98.5 (54)	80 (53.3)	0.09
Triglyceride (mg/dl) (IQR)	130 (86)	97 (35)	**<0.01**
Presence of diabetes (%)	30.4	–	–
Presence of metabolic syndrome (%)	39.1	–	–
Fibrosis (kPA) (IQR)	5.4 (2.6)	4.9 (2.5)	0.29
Controlled attenuation parameter (dB/m) (IQR)	307.5 (76)	300 (116)	0.59
**Ethnicity (%)**			
White (*n* = 19)	41.3	–	–
African American (*n* = 15)	32.6	–	–
Hispanic (*n* = 11)	23.9	–	–
Asian (*n* = 1)	2.2	–	–

*Continuous variables are listed as median with their interquartile range (IQR)*.

*Significant p-values are bolded*.

**Table 2:** Macronutrient intake at baseline and at the final visit as measured by the Diet History Questionnaire III.

	**Baseline (*n* = 46)**	**Final (*n* = 46)**	***p*-value**
Total calories (kcal) (IQR)	2051.3 (1387.4)	1492.9 (965.5)	**<0.01**
Protein (kcal) (IQR)	370.8 (251.6)	282.8 (157.2)	0.11
Fat (kcal) (IQR)	798.3 (600.8)	510.2 (398.0)	**<0.01**
Carbohydrate (kcal) (IQR)	902.7 (614.4)	649.1 (462.0)	**<0.01**

*Values are listed as median with their interquartile range (IQR)*.

*Significant p-values are bolded*.

**Table 3:** Patient characteristics and clinical outcomes comparing study participants who lost at least 5% of their body weight to those that did not.

	**<5% weight change (*n* = 32)**	**>5% weight change (*n* = 14)**	***p*-value**
Gender (% males)	75.0	78.6	1.00
Age (years) (IQR)	57.0 (20.0)	56.5 (9)	0.98
Change in BMI (IQR)	−0.7 (1.5)	−2.3 (0.6)	**<0.01**
Change in weight (kg) (IQR)	−2.0 (3.9)	−7.1 (1.7)	**<0.01**
Change in percent body weight (IQR)	−1.9 (4.1)	−7.5 (1.5)	**<0.01**
Change in HbA1c (IQR)	−0.0 (0.5)	−0.2 (0.3)	0.21
Change in total cholesterol (mg/dl) (IQR)	−8.5 (45)	−2.5 (30)	0.69
Change in HDL (mg/dl) (IQR)	−1.0 (9.7)	3.1 (8.9)	0.23
Change in LDL (mg/dl) (IQR)	−3.5 (41.8)	4.0 (35.0)	0.19
Change in triglyceride (mg/dl) (IQR)	−4.5 (45.5)	−54 (54)	**<0.01**
Change in fibrosis (kPA) (IQR)	0.1 (2.2)	−1.2 (3.1)	**0.04**
Change in controlled attenuation parameter (dB/m) (IQR)	23.5 (72.0)	−65.5 (92)	**<0.01**
**Ethnicity (%)**
White (*n* = 19)	43.8%	35.7%	0.46
African American (*n* = 15)	34.4%	28.6%	
Hispanic (*n* = 11)	21.9%	28.6%	
Asian (*n* = 1)	0.0%	7.1%	

*Significant p-values are bolded*.

**Table 4:** Change in macronutrient intake by study participants who lost at least 5% of their body weight as compared to those that did not.

	**<5% weight change (*n* = 32)**	**>5% weight change (*n* = 14)**	***p*-value**
Change in % total calories (IQR)	−23.7 (44.7)	−28.2 (111.7)	0.89
Change in % protein (IQR)	−8.7 (64.5)	−6.8 (86.0)	0.85
Change in % fat (IQR)	−29.0 (42.5)	−29.4 (72.8)	0.96
Change in % carbohydrate (IQR)	−27.5 (37.6)	−34.1 (164.6)	0.81
Compliant (%)	53.1	57.1	0.99

*Compliance was defined as at least 500 kcal per day reduction in total calorie intake from their baseline as measured by the Diet History Questionnaire III. Values are listed as median with their interquartile range (IQR)*.

**Figure 2:**
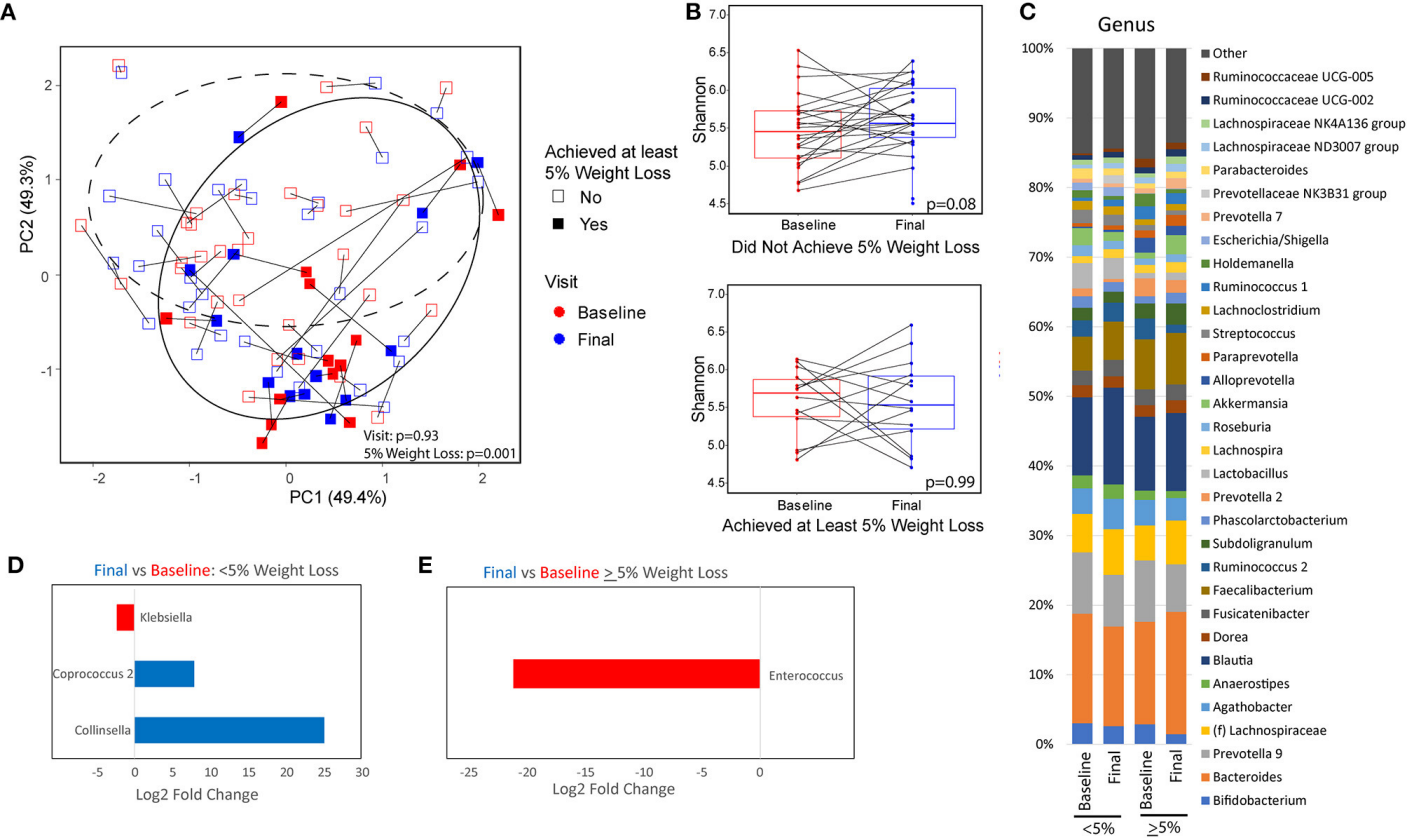
Microbiome differences between baseline and after a calorie-restricted diet by weight loss category. **(A)** Principal coordinate analysis plot using the Robust Aitchison distance metric colored by visit with different shapes and 95% confidence interval ellipses corresponding to the amount of weight loss. Dashed ellipse encircles those that did not achieve at least 5% weight loss. Solid ellipse encircles those that did achieve at least 5% weight loss. **(B)** Microbial diversity as measured by Shannon index (measurement of species richness and evenness) between baseline and end of study, stratified by weight loss. **(C)** Taxonomic summary plots of genera stratified by timepoint and weight loss. Only genera with at least 1% relative abundance are listed. **(D,E)** Genera that are differentially abundant between baseline and week 16 for patients with **(D)** minimal weight loss and those with **(E)** at least 5% weight loss. Red indicates genera that are overabundant at baseline and blue indicates genera that are overabundant at the final visit. Magnitude of difference is represented as the log 2 of the fold change estimated from DESeq2 models.

The fecal microbiome of subjects was assessed by 16S rRNA sequencing at baseline and at 16 weeks. Patients who were able to achieve at least 5% weight loss had significant differences in overall microbial composition (beta diversity) both at baseline and at 16 weeks as compared to those that did not have significant weight loss (*p* = 0.001; Figure 2A). There were no significant differences in overall microbial composition between baseline and end of treatment for either patients who had significant weight loss (*p* = 0.83) or patients who did not have significant weight loss (*p* = 0.93). There were also no significant differences in overall microbial composition at baseline between patients with diabetes vs. those without diabetes (*p* = 0.64) or those who were compliant to the calorie restricted diet vs. those who were non-compliant (*p* = 0.44). Patients who lost <5% of their body weight showed a non-significant trend toward higher alpha diversity by the Shannon index of microbial richness and evenness at the end of the trial as compared to their baseline values (*p* = 0.08; Figure 2B). There was no difference in Shannon index at 16 weeks for patients with significant weight loss as compared to their baseline values. There was also no difference in alpha diversity between patients with or without significant weight loss at either baseline or week 16. The genus level taxonomic profiles of subjects at baseline and week 16 are shown in Figure 2C, stratified by weight loss. Differential abundance testing at the genus level demonstrated significant reduction in *Klebsiella* and enrichment of *Coprococcus* and *Collinsella* while on a calorie-restricted diet in the group that did not have significant weight loss (Figure 2D). The only significant change in the group with at least 5% weight loss was a reduction in *Enterococcus* (Figure 2E).

**Figure 3:**
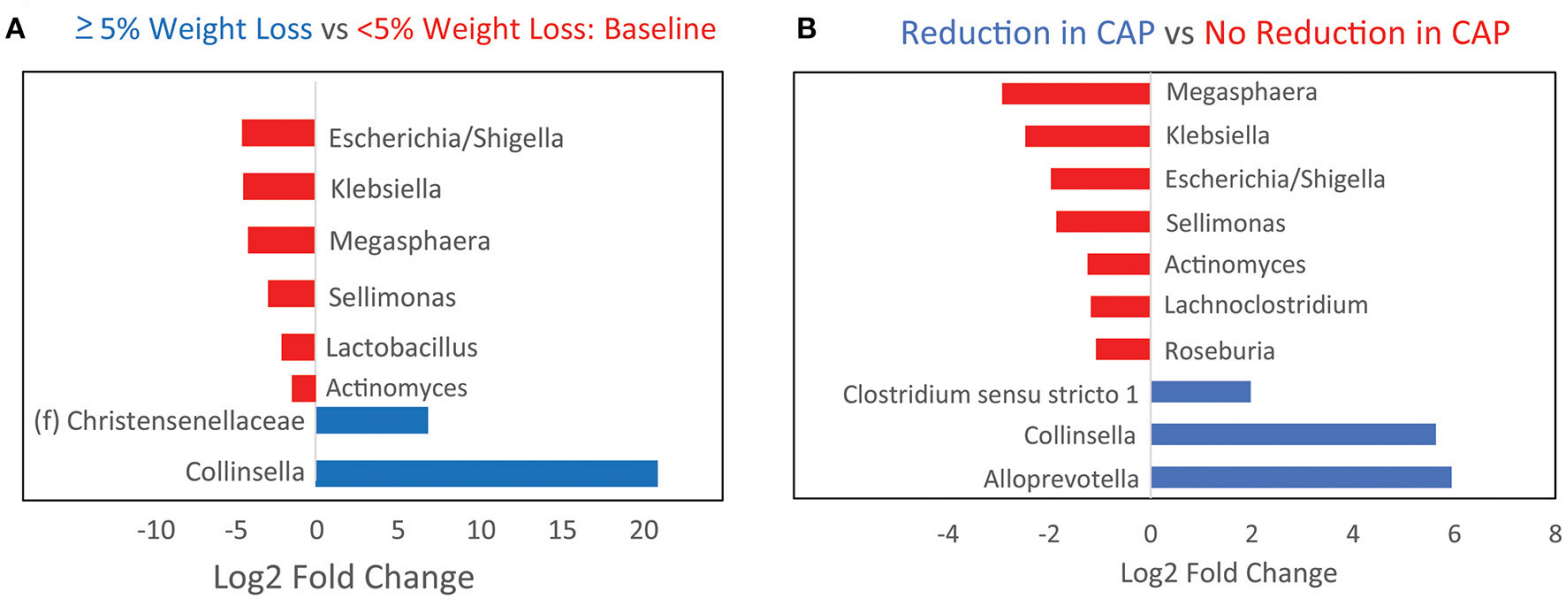
Baseline microbiome differences between patients stratified by whether they achieved at least 5% weight loss or a reduction in hepatic steatosis on a calorie-restricted diet. **(A)** Bar plots showing baseline genera differences from DESeq2 analysis between patients who developed at least 5% weight loss as compared to those that did not. Red indicates genera that are overabundant in patients with <5% weight loss. **(B)** Baseline genera differences between patients who had reduction in hepatic steatosis by CAP score as compared to those that did not. Red indicates genera that are overabundant in patients that did not have any reduction in steatosis.

**Figure 4:**
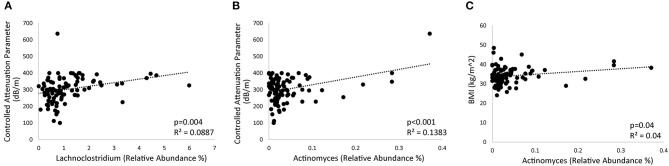
Bacterial genera associated with hepatic steatosis and weight. **(A,B)** Regression plots showing the correlation of **(A)** *Lachnoclostridium* and **(B)** *Actinomyces* with hepatic steatosis as measured by controlled attenuated parameter (CAP). **(C)** Regression plot showing the relationship between *Actinomyces* and BMI.

Further analysis was performed to identify microbial genera that differed at baseline between those that had significant weight loss vs. those that did not and that differed between those that had reduced hepatic steatosis as compared to those that did not. Patients with at least 5% weight loss on a calorie-restricted diet had less *Escherichia/Shigella, Klebsiella, Megasphaera, Sellimonas*, and *Lactobacillus*, and more *Collinsella* and an unidentified genus in the family *Christensenellaceae* as compared to those that did not respond as well to a calorie-restricted diet (Figure 3A). About half of the patients had a reduction in their CAP score at 16 weeks while the other half had no change or elevated levels at 16 weeks. Patients with reduced hepatic steatosis on a calorie-restricted diet as measured by CAP had less *Megasphaera, Klebsiella, Escherichia/Shigella, Sellimonas, Anaerostipes, Lachnoclostridium*, and *Roseburia*, and more *Clostridium sensu stricto, Collinsella*, and *Alloprevotella* as compared to subjects that did not have any reduction in hepatic steatosis (Figure 3B). Linear regression analysis using Spearman's correlation of these differentially abundant microbes showed that the relative abundance of *Lachnoclostridium* and *Actinomyces* were linearly correlated with hepatic steatosis (CAP score) (*p* = 0.004 and *p* <0.001, respectively), while only the relative abundance of *Actinomyces* was linearly correlated with BMI (*p* = 0.04) (Figure 4). A mediation analysis was performed to examine the effect of weight change on hepatic steatosis with the relative abundance of *Actinomyces* as a mediator. While the correlation between *Actinomyces* and BMI to hepatic steatosis was significant, the mediation effect of *Actinomyces* was not (*p* = 0.22).

**Figure 5:**
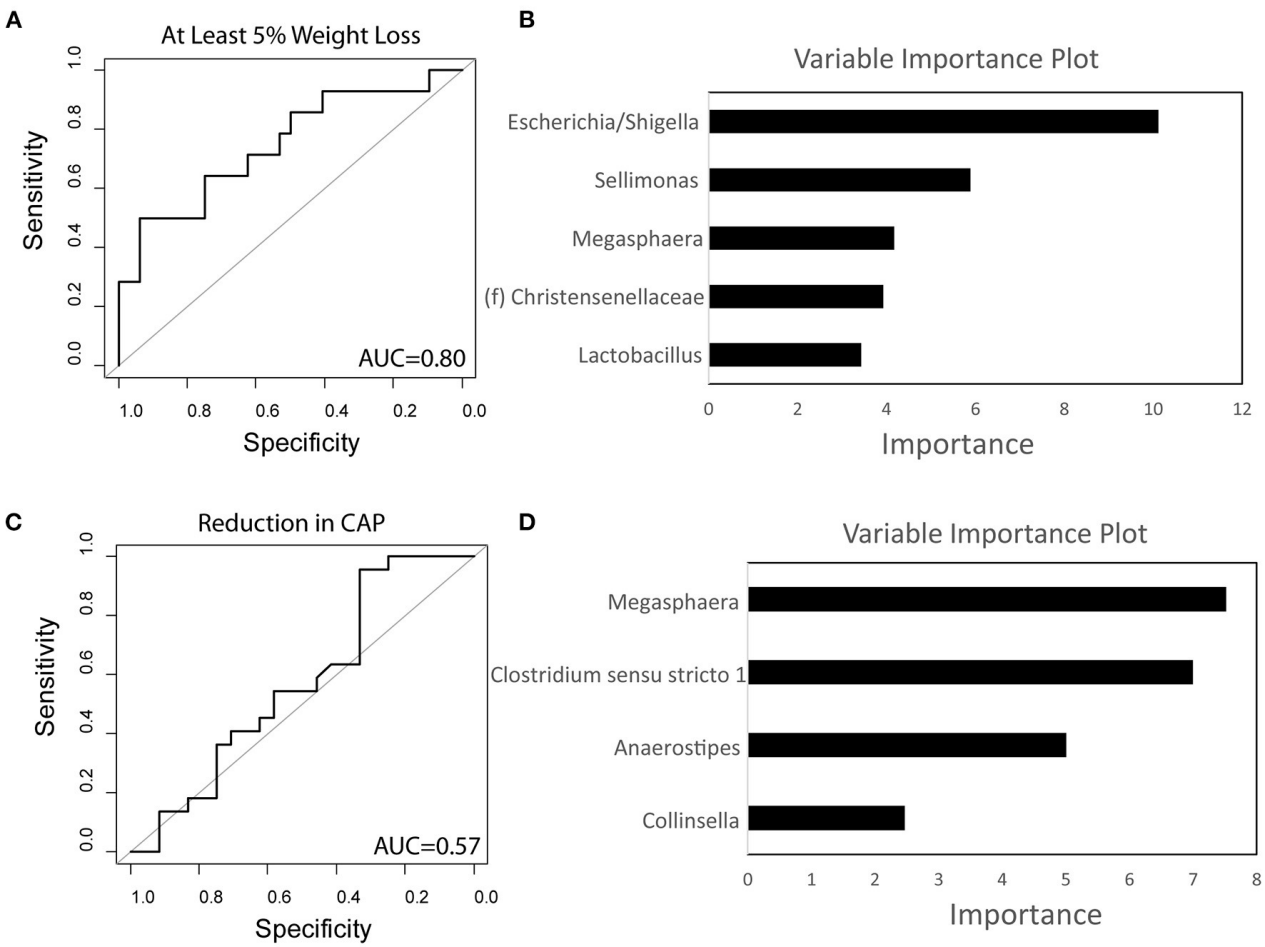
Baseline microbiome predicts weight loss on a calorie-restricted diet. **(A)** Receiver operating characteristic curve of the random forest classifier using baseline microbiome data to predict at least 5% weight loss. **(B)** Variable importance plot of the classifier for weight loss. **(C)** Receiver operating characteristic curve of the random forest classifier using baseline microbiome data to predict reduction in hepatic steatosis as measured by controlled attenuated parameter (CAP). **(D)** Variable importance plot of the classifier for steatosis. AUC, Area under the curve.

Using baseline microbiome data, a random forest classifier was generated to predict those that would have significant weight loss on a calorie-restricted diet and to predict those that would have a reduction in hepatic steatosis while on a calorie-restricted diet. The baseline microbiome was highly accurate at predicting weight loss of at least 5% at 16 weeks for subjects undergoing a calorie-restricted diet with an area under the receiver operating characteristic curve (AUROC) of 0.80 (Figure 5A). The microbes that were most important to this classifier were *Escherichia/Shigella, Sellimonas*, and *Megasphaera* (Figure 5B). The baseline microbiome was less accurate at predicting hepatic steatosis reduction in subjects undergoing a calorie-restricted diet (AUROC = 0.57) (Figure 5C). The microbes that were most important to this classifier were *Megasphaera* and *Clostridium sensu stricto* (Figure 5D).

## Discussion

In this prospective study of overweight and obese subjects undergoing a calorie-restricted diet, we demonstrated that baseline microbiome differences existed between those who experienced at least 5% weight loss compared to those with less response. Not surprisingly, patients who had significant weight loss saw improvements in several metabolic factors including total cholesterol, serum triglyceride, and HbA1c. Similar to previous works, subjects that had significant weight loss also saw significant improvement in their hepatic steatosis and fibrosis as compared to those that had just mild weight loss (31). We also saw that participants without diabetes lost more weight than those with diabetes. This is consistent with other published studies showing resistance to weight loss in patients with insulin resistance (32). Participants who completed the study on average ate fewer calories with a reduction in their intake of fats and carbohydrates, which likely represents reduced intake of processed carbohydrates with high fat content.

This is one of the few studies to examine the longitudinal changes of the gut microbiome in subjects undergoing a calorie-restricted diet. We found that 16 weeks of a macronutrient standardized diet with 14 weeks of calorie restriction had only a modest effect on the gut microbiome. In particular, calorie restriction resulted in reduced abundance of *Klebsiella* and *Enterococcus*, which have both been associated with obesity and MAFLD (33, 34). For example, in a Chinese cohort, researchers found *Klebsiella* with high alcohol production was present in 60% of patients with MAFLD (33). When introduced into mice, this specific strain was able to induce fatty liver disease (33). Furthermore, *Klebsiella* has also been shown to reduce gut barrier function, which is a hallmark of both obesity and MAFLD (35, 36).

While the changes in the gut microbiome after calorie restriction were modest, there was significant differences in baseline microbiome of patients that responded to a calorie-restricted diet vs. those that did not. Similar differences were observed when comparing those who had hepatic steatosis improvement vs. those that did not, reflecting the strong correlation between weight loss and improvement in hepatic steatosis. *Escherichia/Shigella, Klebsiella, Megasphaera, Actinomyces* were overabundant in patients that did not have significant weight loss or hepatic steatosis improvement. Similar to *Klebsiella, Escherichia/Shigella, Megasphaera*, and *Actinomyces* have each been associated with obesity (37–39). In particular, *Escherichia/Shigella* has been associated with insulin resistance and type 2 diabetes (38). Therefore, the presence of these microbes may mark individuals with a more resistant form of metabolic disorder that is less likely to be affected by calorie restriction alone. We also found that *Lachnoclostridium* was linearly related to hepatic steatosis. In an animal model of fatty liver disease, *Lachnoclostridium* was also positively associated with obesity and hepatic steatosis (40). Whether these microbiome changes are pathological or merely associations should be the focus of future research.

One of the major findings of this study was that a classifier based on baseline microbiome data was able to accurately predict weight loss for subjects undergoing a calorie-restricted diet. This suggests that the microbiome at baseline can be used as a biomarker to predict those who are likely to respond to life-style modifications. Surprisingly, the rate of compliance with a calorie restricted diet did not differ between patients who lost at least 5% of their body weight as compared to those that did not. Patients who lost more weight did not significantly differ in calorie intake. This suggests that significant weight loss is not entirely dependent on compliance with a calorie restricted diet or the number of calories reduced. The significant association of the intestinal microbiome with patient response suggests that the microbiome may play a role in modulating the metabolic consequences of calorie restriction. Patients with a microbiome less likely to lead to weight loss on a calorie-restricted diet may potentially benefit more from other forms of weight management such as bariatric surgery or endoscopic bariatric surgery. In addition, our findings suggest that altering a subject's baseline microbiome through strategies that may decrease *Escherichia/Shigella, Klebsiella, Megasphaera*, and/or *Actinomyces* may increase their likelihood of clinical response to a calorie-restricted diet.

The study has several limitations. Even though patients were monitored and counseled regularly on calorie restriction, the proportion of patients that lost at least 5% of their body weight was relatively low with only one patient being able to reach at least 10% weight reduction. Therefore, more significant effects of calorie restriction on the gut microbiome may have been seen if more patients had achieved significant weight loss. Also, the microbiome changes seen between the 14 patients that achieved significant weight loss vs. the others that did not may be due to sampling bias. Therefore, larger studies will need to be done to confirm the findings presented here. Moreover, while we counseled patients to not increase or decrease their physical activity level during the trial, we did not measure physical activity directly. We cannot exclude effects of altered physical activity on some of the outcomes measured in this study. Furthermore, while the longitudinal design of this study is a strength, the findings are still associative. Mechanistic studies using animal models will be needed to explore the mechanistic basis for associations of microbial profiles with response to calorie-restricted diets. Additionally, due to the small sample size, we were unable to split the cohort into a derivation and validation cohort. While we assessed classifier performance using *k*-fold cross validation, the classifier predicting weight loss will still need to be validated in an external cohort. Finally, the relatively high dropout rate may have introduced attrition bias and may have confounded results. However, we postulate that this may be negligible as the vast majority of patients who dropped from the study did so within 2–4 weeks.

In conclusion, calorie restriction is associated with intestinal microbiome changes and improvement in hepatic steatosis. Several genera in the gut microbiome are associated with both weight and hepatic steatosis. Baseline microbiome differences predict who will likely lose significant weight in response to a calorie-restricted diet, suggesting a role of the gut microbiome in mediating clinical response to calorie restriction.

## Data Availability Statement

The datasets presented in this study can be found in online repositories. The names of the repository/repositories and accession number(s) can be found below: NCBI SRA BioProject, Accession: PRJNA736811.

## Ethics Statement

The studies involving human participants were reviewed and approved by Greater Los Angeles Veterans Affair Hospital. The patients/participants provided their written informed consent to participate in this study.

## Author Contributions

JJ, TD, JP, and ZL: conceptualization. TD, VL, S-LW, and ZL: methodology. TD, VL, and JYa: software. TD and JJ: validation, writing—original draft preparation, writing—review, and editing. TD: formal analysis and visualization. TD, KL, VL, FS, S-LW, BD, WK, CC, YZ, NA-J, JYe, AA, and JJ: investigation. JJ, JP, and ZL: resources. TD, KL, and VL: data curation. JJ: supervision, project administration, and funding acquisition. All authors have read and agreed to the published version of the manuscript.

## Conflict of Interest

The authors declare that the research was conducted in the absence of any commercial or financial relationships that could be construed as a potential conflict of interest.
